# Effects of Huazhuo Jiedu Shugan Decoction on Cognitive and Emotional Disorders in a Rat Model of Epilepsy: Possible Involvement of AC-cAMP-CREB Signaling and NPY Expression

**DOI:** 10.1155/2019/4352879

**Published:** 2019-12-13

**Authors:** Xin Ping, Shao-Kun Qin, Shu-Ning Liu, Ye Lu, Ya-Nan Zhao, Ya-Fei Cao, Yan-Hong Zhang, Shao-Dan Zhang, Li Chu, Lin Pei

**Affiliations:** ^1^School of Chinese Medicine, Hebei University, Baoding, Hebei 071051, China; ^2^School of Chinese Medicine, North China University of Science and Technology, Tangshan, Hebei 063210, China; ^3^Hebei Key Laboratory of Turbidity, Hebei Academy of Chinese Medicine Sciences, Shijiazhuang, Hebei 050011, China; ^4^Hebei University of Chinese Medicine, Shijiazhuang, Hebei 050051, China; ^5^The Second Hospital of Hebei Medical University, Shijiazhuang, Hebei 050073, China

## Abstract

**Background:**

Huazhuo Jiedu Shugan decoction (HJSD), a traditional Chinese medicine (TCM), has been used to treat epileptic seizures for many years. Some ingredients in these herbs have been demonstrated to be effective for the treatment of brain damage caused by epilepsy.

**Aim of the Study:**

The object of the study is to determine the effects of HJSD on cognitive and emotional disorders in a rat model of epilepsy.

**Materials and Methods:**

After a predetermined time period, rats were intraperitoneally injected with pentylenetetrazol and observed in different phases of convulsions. The cognitive and emotional changes in the epileptic rats were assessed using behavioral and immunohistochemical tests.

**Results:**

Compared with the epilepsy group, the seizure grade was reduced and seizure latency was prolonged following HJSD-H treatment (*P* < 0.01). Compared with the control group, the epilepsy group displayed marked worse performance on the animal behavior tests (*P* < 0.05) and the HJSD-H group displayed improved behavioral performance (*P* < 0.05). After HJSD-H treatment, the expression of adenylate cyclase (AC), cyclic adenosine monophosphate (cAMP), cAMP-response element binding protein (CREB), and neuropeptide Y (NPY) immunoreactive cells markedly increased in the hippocampus, compared with that of the epilepsy group (*P* < 0.05).

**Conclusions:**

The current results demonstrate that HJSD treatment in epileptic rats markedly inhibits epileptic seizures and improves cognitive and emotional disorders, which may be related to the regulation of AC-cAMP-CREB signaling and NPY expression in the hippocampus. The effects of the HJSD treatment may provide a foundation for the use of HJSD as a prescription medicinal herb in the TCM for the treatment of epilepsy.

## 1. Introduction

An epileptic seizure is a transient occurrence of signs and/or symptoms due to abnormal excessive or synchronous neuronal activity in the brain. Epilepsy is a disorder of the brain characterized by an enduring predisposition to generate epileptic seizures and by the neurobiological, cognitive, psychological, and social consequences of this condition [1]. In addition to a shortened life span or increasing the risk of sudden and accidental death, patients with intractable epilepsy also have marked cognitive impairments, emotional disorders, and psychosocial problems, such as restricting employment, reducing marriage rate, and reducing quality of life [2].

The cognitive impairments and emotional disorders caused by epilepsy are related to abnormal neuronal discharge, neuron dysfunction, synaptic recombination, and disorders caused by epilepsy. The cognitive impairments associated with epilepsy seriously influence the quality of life in patients, and about 50% of patients with epilepsy have varying degrees of cognitive impairment. Complex partial epilepsy, temporal lobe epilepsy, generalized convulsive epilepsy, and even absence epilepsy can all result in cognitive impairments [3]. Epilepsy patients are often accompanied by emotional disorders, including depression, anxiety, fear, and so on [4]. Some studies have shown that the cyclic adenosine monophosphate (cAMP) signaling pathway is closely related to epileptic seizures [5], and the role of neuropeptide Y (NPY) in antiepileptic has also been demonstrated [6, 7]. Experimental epilepsy models, such as chemical kindling, have played a key role in reforming our understanding of the related mechanisms of epileptic seizures. In the kindling model of epilepsy, repetitive application of an initially subconvulsive chemical stimulation results in stable neuronal and synaptic changes in various areas of the brain. More detailed studies regarding the influence of antiepileptic drugs on kindling may provide a better understanding of the antiepileptic mechanisms [8].

To date, first-line antiepileptic drugs (AEDs) have taken precedence in the treatment of epileptic seizures. Long-term application of AEDs, however, can cause resistance to these drugs, decreasing their clinical effects, or causing cognitive impairments and emotional disorders [9]. Research has shown that the use of traditional Chinese medicine (TCM) in epilepsy may be beneficial because these techniques cause few adverse effects compared to mainstream pharmaceuticals, thus providing a novel method of epileptic therapy [10]. TCM has a long history and has been widely used in the prevention and treatment of epilepsy in China [11]. Studies have shown that TCM herbs can be an effective therapeutic strategy for treating epilepsy patients and to aid the development of novel drugs [12]. Our clinical observations have shown that Huazhuo Jiedu Shugan decoction (HJSD, i.e., modified Tiaogan Jiedu recipe) is a feasible treatment strategy for epilepsy, especially for improving epileptic seizures and neurological function in epileptic patients [13]. Some ingredients in HJSD (Table 1) have been demonstrated to be effective for the treatment of brain damage caused by epilepsy [14–17]. In this study, the antiepileptic effect of HJSD and its effect on behavior were observed in epileptic rats, and the possible mechanism(s) of action was explored.

## 2. Materials and Methods

### 2.1. Experimental Animals

Sixty male Sprague Dawley rats (4-week-old, 80–120 g) were used in the study. Rats were acquired from the Animal Experiment Center of Hebei Medical University in Hebei, China (license number SCXK 2018-004 and certificate number 1808129). Three rats were housed per polysulfone cage at ambient temperature (22–25°C) and relative humidity (60 ± 5%). Animals were free-fed a rat pellet artificial diet (Animal Experiment Center of Hebei Medical University, Hebei, China, license number SCXK 2018-003) and tap water. The rats were adapted to laboratory conditions 7 days before experimentation.

All experiments were conducted between 8:00 AM and 2:00 PM, and all animal experiments were conducted according to the guidelines of the National Institutes of Health's Guide for the Care and Use of Laboratory Animals. All efforts were made to minimize the suffering of animals and reduce the number of animals used. All experimental procedures were carried out after being approved by the Laboratory Animal Administration and Ethics Committee of Hebei Academy of Chinese Medicine Sciences (approved number: DWLL2018001 and date: Jun. 22, 2018).

### 2.2. Drugs and Other Chemicals

The total amount of crude drugs in HJSD is 60 g, among which the drug composition and dose are *Scutellaria baicalensis* (Huang qin) 12 g, Gynostemma pentaphylla (Jiao gu lan) 12 g, Radix bupleuri (Chai hu) 12 g, Rhizoma acori graminei (Shi chang pu) 9 g, lotus petiole (He geng) 9 g, and basil (Luo le) 6 g.

The HJSD and sodium valproate tablets (VPA, Hunan Xiangzhong Pharmaceutical Co. Ltd.) were purchased from the Affiliated Hospital of Hebei Academy of Chinese Medicine Sciences. Pentylenetetrazol (PTZ, P6500-25G, Sigma-Aldrich), anti-AC rabbit polyclonal antibody (TA322232, Origene), rabbit anti-cAMP polyclonal antibody (TA327118, Origene), CREB (48H2) Rabbit mAb (#9197, Cell Signaling Technology), and anti-NPY antibody (ab30914, Abcam) were purchased from Hebei Bio-high Technology Co. Ltd., China. All other chemicals were sourced from the Hebei Academy of Chinese Medicine Sciences and were of analytical grade.

### 2.3. Groups and Drug Administration

The rats were randomly divided into six groups (10 rats in each group) as follows: the control group, the epilepsy group, the VPA group (300 mg/kg/day), the HJSD high-dose group (HJSD-H, 4.32 g/kg/day), the HJSD medium-dose group (HJSD-M, 2.16 g/kg/day), and the HJSD low-dose group (HJSD-L, 1.08 g/kg/day). The volume for HJSD treatment (low, medium, or high dose) was 5 ml/kg. HJSD and VPA were administered by gastric gavages between 9:00 AM and 10:00 AM during the treatment period. These doses are generally consistent with and cover the clinical dose of HJSD and the intragastric dose of the animals referred to in previous animal studies [18]. VPA of intragastric administration was performed according to the study by Cheng et al. [19].

### 2.4. Experimental Scheme

**Figure 1:**
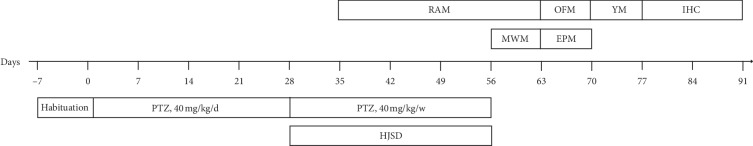
Timeline of the experiment. Habitual feeding was conducted 7 days before the formal experiment. Epilepsy was induced by intraperitoneal injection of PTZ for 28 days. The model was maintained once per week for the next 28 days. The drug administration began on day 28 and continued for 28 days. The RAM test was performed from day 35 until day 63, the MWM test was performed from day 56 until day 63, the OFM and EPM tests were conducted from day 63 until day 70, the YM test was performed from day 70 until day 77, and immunohistochemistry was performed from day 56 until day 70.

An epilepsy model induced by PTZ was used in this study. Seizure grade and incubation period, animal behaviors, and immunohistochemical staining were also evaluated. The experimental design is illustrated in Figure 1.

### 2.5. PTZ Kindling

The modified PTZ kindling model was established by intraperitoneal injections (IP) of a subconvulsant dose of PTZ once daily until complete kindling was achieved in rats for 28 consecutive days [20, 21]. For the kindling epileptic model, the rats were injected with PTZ (40 mg/kg, IP) each day (24 ± 1 h). The rats were then placed in polysulfone cages and monitored for 40 min. Seizure activity was recorded using a stage of 0 to 7 [22]. The behavioral seizure scale in rat pups may be summarized as follows: Stage 0, behavioral arrest; Stage 1, mouth clonus; Stage 2, head bobbing; Stage 3, unilateral forelimb clonus; Stage 3.5, alternating forelimb clonus; Stage 4, bilateral forelimb clonus with rearing; Stage 5, bilateral forelimb clonus with rearing and falling over; Stage 6, wild running and jumping with vocalization; and Stage 7, tonus. The rats showing Stage 3.5 or higher seizures were considered kindled. If the rats were not kindled within 10 minutes, an additional PTZ dose of 20 mg/kg was administered and the rats were observed for 10 minutes. If the rats were still not kindled, an additional dose of 10 mg/kg PTZ was administered every 10 minutes until the animal was kindled. A maximum of 30 injections of PTZ was given (according to the method shown by Joshi et al.) or until the development of kindling, whichever was earlier [23]. In order to avoid death caused by convulsive symptoms during epileptic seizures in epileptic rats, chloral hydrate (0.3 ml/100 g, IP) was used for sedation and anticonvulsion [24].

### 2.6. Animal Behaviors

All animal behaviors were tested and documented using the SLY-ETS animal behavioral activity record analysis system (version 1.66, Beijing Shuolinyuan Technology Co. Ltd.).

#### 2.6.1. Morris Water Maze Test

The Morris water maze (MWM) test was performed to measure learning and memory in epileptic rats. The experiment was conducted according to the methods described in Cao et al. [25]. Briefly, the test included place navigation and a spatial probe test. At the beginning of the experiment, the rats were artificially placed in the water facing the wall, and the experiment was terminated when the rats reached the platform. The rats were given 120 s to search the hidden platform. Before the next experiment, all the rats were placed on the platform for 15 seconds. During the first four days of the experiment, we observed and recorded the latency of rats while searching for hidden platforms. On the last day, probe tests without the hidden platform were evaluated at a different starting point and the cross frequency of the platform in the target quadrant was recorded.

#### 2.6.2. Radial Arm Maze Test

Briefly, the radial arm maze (RAM) was designed to assess spatial learning and memory in rats. According to the method of Wang et al., before the RAM test, the rats were habituated [26]. In order to ensure sufficient motivation for this test, each rat was starved for 24 h the day before the test (or lost 80% of their regular diet). The test was recorded for a total of 3 min, and the feeding arms (1, 2, 4, and 7) remained baited for habituating trials. The time consumed for four pellets was recorded as the “residence time of correct feeding arms,” and the frequency of entries into unbaited arms was recorded as “reference memory errors.”

#### 2.6.3. Y-Maze Test

The Y-maze (YM) was mainly used to assess discrimination learning, working memory, and the reference memory test of rats according to the method of Mohammadi et al. [27]. Briefly, the YM is based on the natural tendency for rats to explore unfamiliar environments. The rats were placed at the end of the YM starting arm, and the number of evasive errors was recorded by the shock-avoidance test. The experiment was performed five times clockwise. To avoid the occurrence of olfactory effects, YM was completely cleaned with 75% ethanol between tests.

#### 2.6.4. Elevated Plus Maze Test

The elevated plus maze (EPM) measured the anxiety of the rats based on the contrast between exploratory behavior and the tendency to avoid open, well-lit, and elevated spaces as described previously. The test was conducted according to the method described in Marques-Carneiro et al. [28]. Briefly, the rats were brought from the home cage to the laboratory 5 min before they started to adapt to the environment. To begin the test, the rats were placed in the center of the device with their heads facing a constant open arm. The rats were free to explore the device for 5 min. The parameters measured were the number of entries into the open arms and the time spent in open arms. Between each test, the equipment was completely cleaned with 75% ethanol.

#### 2.6.5. Open Field Maze Test

Behavior using the open field maze (OFM) test evaluated spontaneous activity and emotionality. Less activity in the OFM test is thought to represent a higher level of anxiety and vice versa. According to the method of Fan et al., undisturbed rats were recorded by a camera placed above the center of the OFM for 10 minutes [29]. The rats were placed in the center of the OFM, and the time spent in center, the distance of total traveled and central traveled, and the number of times crossed through the central area were recorded. After each rat finished the test and was removed from the area, the device was cleaned with 75% ethanol.

### 2.7. Immunohistochemistry

All rats were sacrificed 28 days after being considered kindled, and the last PTZ administration (40 mg/kg, IP) was made the day before the rats were killed (*n* = 3 for each group). Brain tissue sections were deparaffinized in xylene, rehydrated in a graded series of ethanol for 5 min, and then incubated with H_2_O_2_ (0.3%, 30 min). For antigen retrieval, the sections were heated in a microwave oven with 10 mmol/L sodium citrate buffer (pH 6.0) for 10 min at 92–98°C. The sections were then incubated with goat serum (Zhongshan Golden Bridge, Beijing, China) for 30 min at room temperature [30]. The tissue sections were incubated with anti-AC rabbit polyclonal antibody (1 : 200), anti-cAMP polyclonal antibody (1 : 300), CERB rabbit antibody (1 : 1000), and anti-NPY antibody (1 : 1000) overnight at 4°C. The next day, the tissue sections were incubated with secondary goat anti-rabbit antibody for 30 min at 37°C and were then treated with an avidin-biotin complex (ABC) solution (Zhongshan Golden Bridge, Beijing, China) for 30 min. The sections were washed with phosphate-buffered saline (PBS) and then incubated with 3,3-diaminobenzidine (DBA) (Zhongshan Golden Bridge) for 3 min before hematoxylin was used for counterstaining. For negative controls, PBS was used instead of the primary antibodies; all other procedures were the same as those described above. An Olympus CX41 (Olympus, Osaka, Japan) automatic microscope was used to examine the images. Five visual field images were randomly obtained from each section. Cells in the cytoplasm or cell membrane were considered positive for adenylate cyclase (AC), cAMP, cAMP-response element binding protein (CREB), and NPY. Finally, we used Image-Pro plus 5.1 software (Media Cybemetrics, USA) to calculate the mean OD of each vision field and to evaluate the immunopositivity of the images.

### 2.8. Statistical Analysis

Data are presented as the mean ± SEM. All data were analyzed with one-way analysis of variance (ANOVA) and the LSD method test. All statistical analyses were performed using SPSS 23.0 software (IBM, Chicago, IL, USA). *P* < 0.05 was considered to be statistically significant.

## 3. Results

### 3.1. Effects of PTZ on Rats

The behavioral seizure grade was assessed in the epileptic rat model. During the administration of PTZ, the number of rats killed was 4 and the survival rate of the rats was 92.6% (*n* = 54). The kindled epileptic rats were arbitrarily divided into 5 groups. The drug-resistant animals did not appear in this study.

### 3.2. Effects of HJSD on Epileptic Seizures

**Figure 2:**
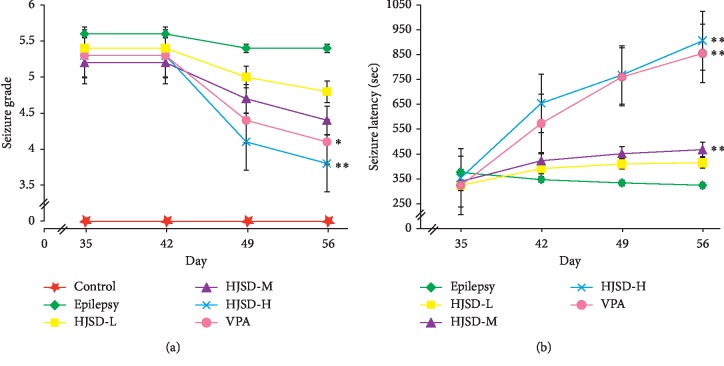
Behavioral effects of HJSD on the seizure grade (a) and seizure latency (b). A statistical significance test was performed using a one-way ANOVA; *n* = 10; ^*∗*^*P* < 0.05, ^*∗∗*^*P* < 0.01, ^*∗*^Comparison with the epilepsy group.

As shown in Figure 2, compared to the epilepsy group, the seizure grade (*F*_4,45_ = 2.704, *P* < 0.01) was reduced while the seizure latency (*F*_4,45_ = 63.772, *P* < 0.01) was prolonged in rats of the HJSD-H group after therapy.

### 3.3. Effects of HJSD on Animal Behavior

#### 3.3.1. Effects of HJSD on the MWM Test

**Figure 3:**
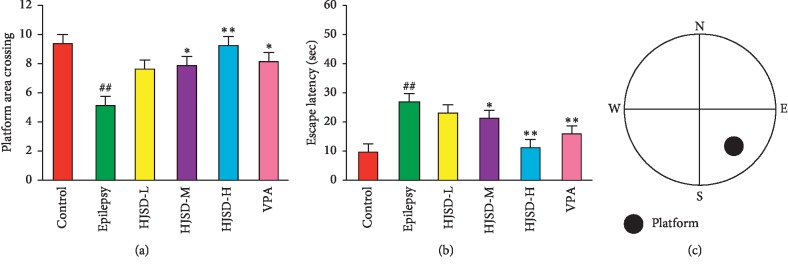
Behavioral effects of HJSD on the MWM test. Times across the platform (a). Escape latency (b). The platform was centered at the southeast quadrant (c). A statistical significance test (for comparison) was performed using a one-way ANOVA; *n* = 8; ^*∗*^*P* < 0.05, ^*∗∗*^*P* < 0.01, ^##^*P* < 0.01, ^*∗*^Comparison with the epilepsy group; ^#^Comparison with the control group.

As shown in Figure 3, compared to the control group, the platform area crossing times (*F*_5,42_ = 2.591, *P* < 0.01) was reduced, and the escape latency (*F*_5,42_ = 16.048, *P* < 0.01) was longer in rats from the epilepsy group. Compared to the epilepsy group, the platform area crossing times was longer, and the escape latency was shortened in rats from the HJSD-H group (*P* < 0.01 and *P* < 0.01, respectively).

#### 3.3.2. Effects of HJSD on the RAM Test

**Figure 4:**
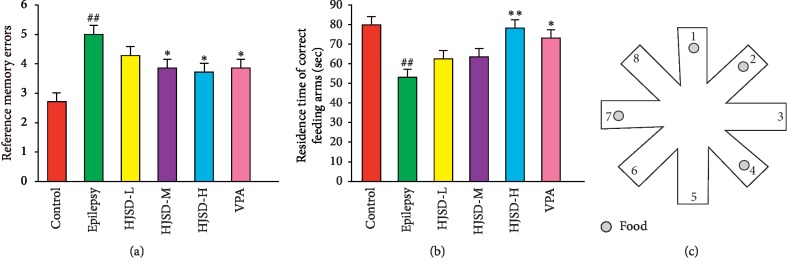
Behavioral effects of HJSD on the RAM test. Reference memory errors (a). Residence time on the correct feeding arms (b). A food with a small pellet at the end of four arms (c). Statistical significance test was determined using a one-way ANOVA; *n* = 7; ^*∗*^*P* < 0.05, ^*∗∗*^*P* < 0.01, ^##^*P* < 0.01, ^*∗*^Comparison with the epilepsy group; ^#^Comparison with the control group.

As shown in Figure 4, compared to the control group, reference memory errors (*F*_5,36_ = 5.045, *P* < 0.05) increased while the residence time of the correct feeding arms (*F*_5,36_ = 2.812, *P* < 0.01) was shortened in rats from the epilepsy group. Compared to the epilepsy group, reference memory errors were reduced, and the residence time on the correct feeding arms increased in rats from the HJSD-H group (*P* < 0.05 and *P* < 0.01, respectively).

#### 3.3.3. Effects of HJSD in YM Test

**Figure 5:**
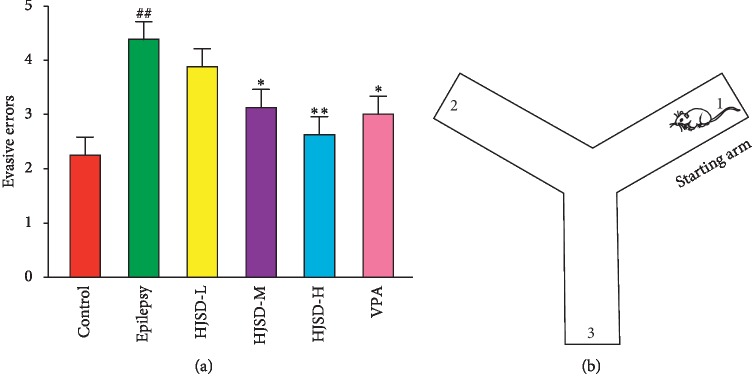
Behavioral effects of HJSD on the YM test. Evasive errors (a). Each test starts with the starting arm (b). Statistical significance was determined using one-way ANOVA; *n* = 8; ^*∗*^*P* < 0.05, ^*∗∗*^*P* < 0.01, ^##^*P* < 0.01, ^*∗*^Comparison with the epilepsy group; ^#^Comparison with the control group.

As shown in Figure 5, compared to the control group, the number of evasive errors was greater in rats from the epilepsy group (*F*_5,42_ = 1.074, *P* < 0.01). After the HJSD-H therapy, the number of evasive errors (*F*_5,42_ = 5.081, *P* < 0.01) was reduced.

#### 3.3.4. Effects of HJSD on the EPM Test

**Figure 6:**
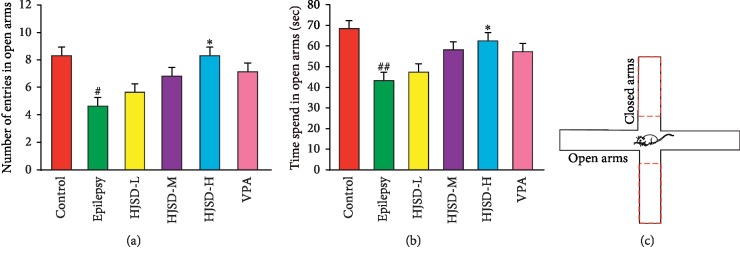
Behavioral effects of HJSD on EPM test. Number of entries into the open arms (a). Time spent in the open arms (b). Rats face the same open arm before the test begins (c). Statistical significance was determined using one-way ANOVA; *n* = 6; ^*∗*^*P* < 0.05, ^#^*P* < 0.05, ^##^*P* < 0.01, ^*∗*^Comparison with the epilepsy group; ^#^Comparison with the control group.

As shown in Figure 6, compared to the control group, the number of entries into the open arms (*F*_5,30_ = 1.396, *P* < 0.05) was reduced among rats in the epilepsy group. Compared to the epilepsy group, the number of entries into the open arms was increased in rats in the HJSD-H group (*P* < 0.05). Compared to the control group, the time spent in the open arms was shortened in rats from the epilepsy group (*F*_5,30_ = 2.307, *P* < 0.01). HJSD markedly prolonged the time spent in the open arms for the epilepsy rats (*P* < 0.05).

#### 3.3.5. Effects of HJSD on the OFM Test

**Figure 7:**
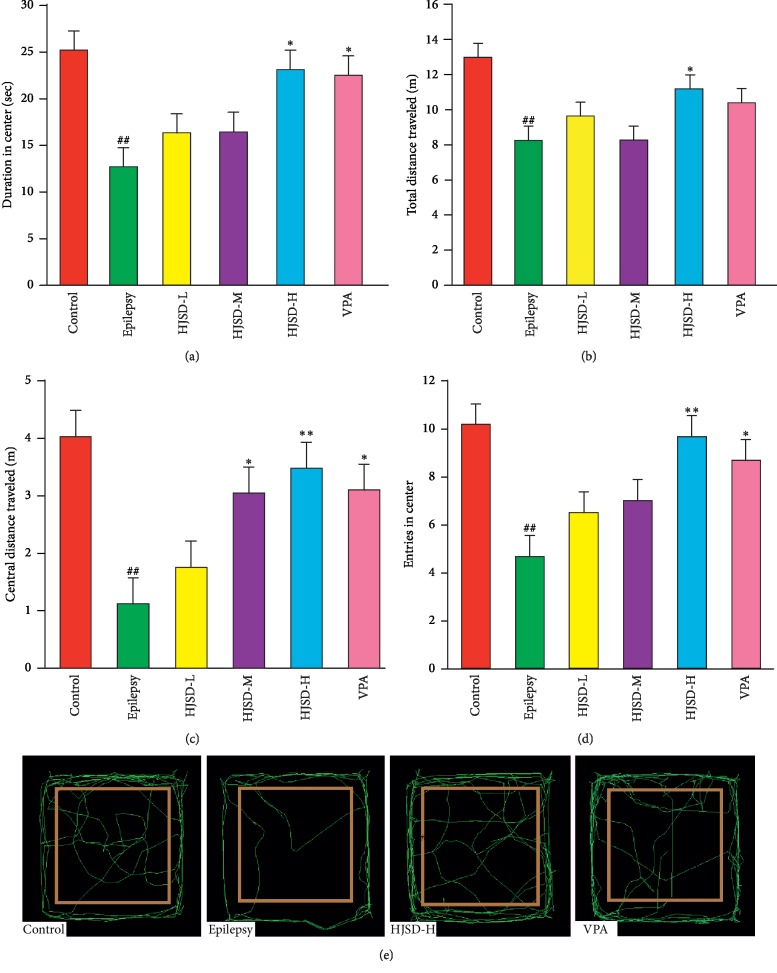
Behavioral effects of HJSD on the OFM test. Duration in the center (a). Total distance traveled (b). Central distance traveled (c). Entries in the center (d). Graphic representation of the OFM movement (e). Statistical significance was determined using one-way ANOVA; *n* = 6; ^*∗*^*P* < 0.05, ^*∗∗*^*P* < 0.01, ^##^*P* < 0.01, ^*∗*^Comparison with the epilepsy group; ^#^Comparison with the control group.

As shown in Figure 7, compared to the control group, the distance of total traveled (*F*_5,30_ = 3.642, *P* < 0.01) and central traveled (*F*_5,30_ = 3.455, *P* < 0.01), the duration (*F*_5,30_ = 2.933, *P* < 0.01), and entries into the center (*F*_5,30_ = 3.692, *P* < 0.01) were reduced in rats form the epilepsy group. The distance of total traveled and central traveled were lengthened, the duration was prolonged, and the number of entries into the center of the OFM increased for rats in the HJSD-H group compared to those in the epilepsy group (*P* < 0.05, *P* < 0.01, *P* < 0.05, and *P* < 0.01, respectively).

### 3.4. Effects of HJSD on Immunoreactive Cell Expression in the Hippocampus

**Figure 8:**
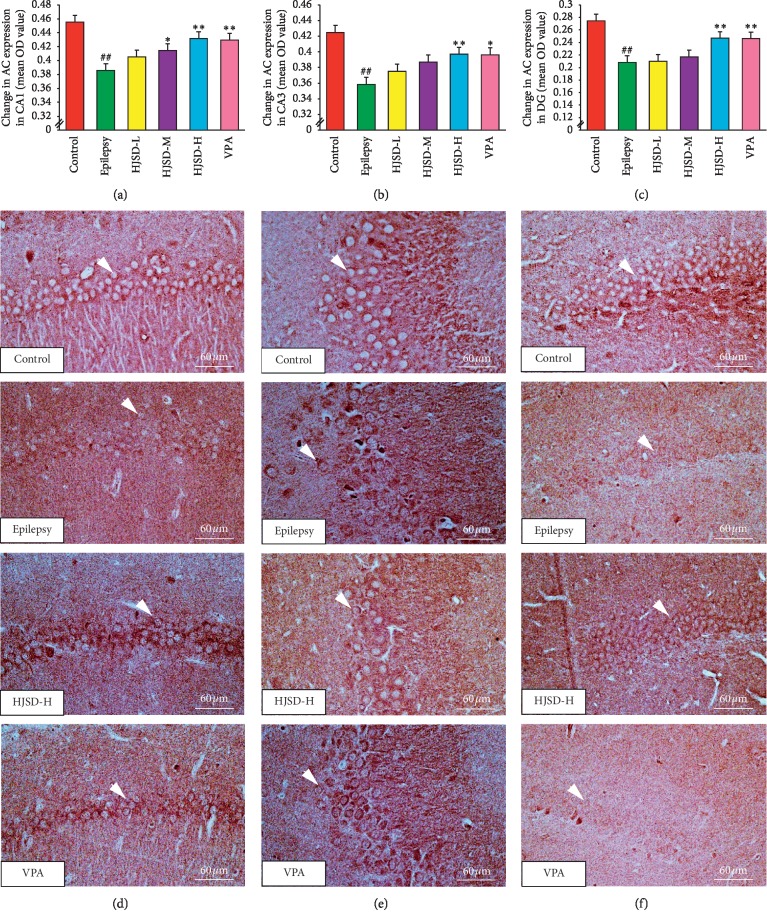
HJSD increases the expression of AC in rat hippocampal CA1 (a), CA3 (b), and DG (c) regions *in vivo*. Number of AC-like immunoreactive cells in the hippocampal CA1 (d), CA3 (e), and DG (f) regions. Statistical significance was determined using one-way ANOVA; *n* = 3; ^*∗*^*P* < 0.05, ^*∗∗*^*P* < 0.01, ^##^*P* < 0.01, ^*∗*^Comparison with the epilepsy group; ^#^Comparison with the control group. Positive expression of AC (arrows) is indicated.

As shown in Figure 8, the expression of AC in CA1 neurons in the epileptic group was markedly reduced compared to that of the control group (*F*_5,444_ = 6.557, *P* < 0.01). After treatment with HJSD-H, the expression of AC markedly increased (*P* < 0.01). In CA3 neurons, the expression of AC in the epileptic group was decreased compared to the control group (*F*_5,444_ = 4.788, *P* < 0.01). After treatment with HJSD-H, the expression of AC was found to be markedly increased (*P* < 0.01). In DG neurons, the expression of AC in the epileptic group was markedly decreased compared to that of the control group (*F*_5,444_ = 7.129, *P* < 0.01). After treatment with HJSD-H, the expression of AC markedly increased (*P* < 0.01).

**Figure 9:**
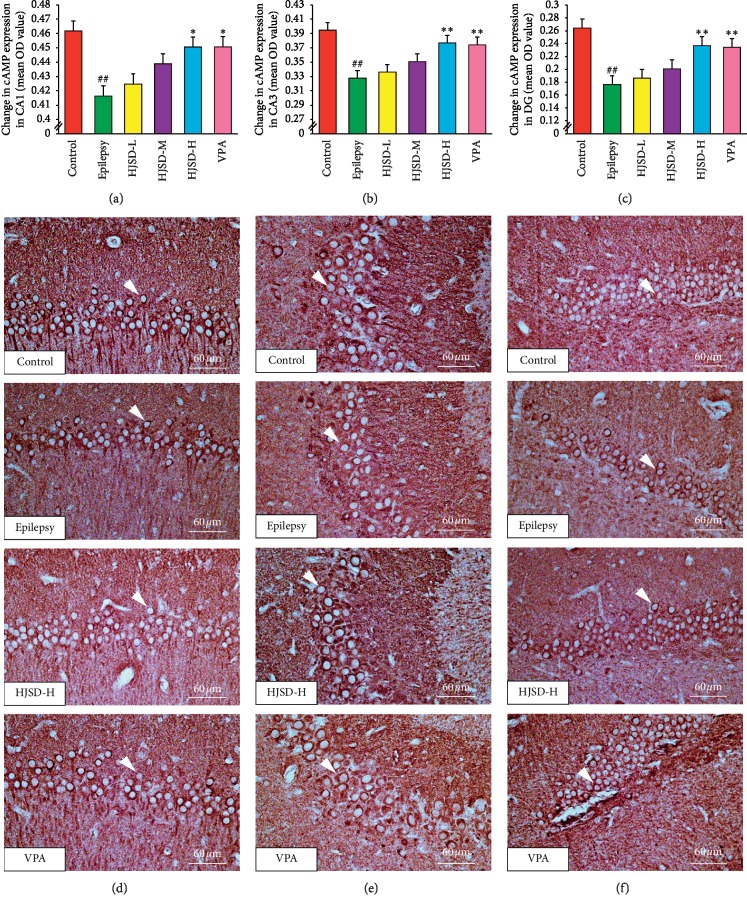
HJSD increases the expression of cAMP in rat hippocampal CA1 (a), CA3 (b), and DG (c) regions *in vivo*. Number of cAMP-like immunoreactive cells in the hippocampal CA1 (d), CA3 (e), and DG (f) regions. Statistical significance was determined using one-way ANOVA; *n* = 3; ^*∗*^*P* < 0.05, ^*∗∗*^*P* < 0.01, ^##^*P* < 0.01, ^*∗*^Comparison with the epilepsy group; ^#^Comparison with the control group. Positive expression of cAMP (arrows) is indicated.

As shown in Figure 9, the expression of cAMP in CA1 neurons in the epileptic group was reduced compared to that of the control group (*F*_5,444_ = 3.090, *P* < 0.01). After treatment with HJSD-H, the expression of cAMP was found to increase (*P* < 0.05). In CA3 neurons, the expression of cAMP was reduced in the epileptic group compared to that of the control group (*F*_5,444_ = 5.565, *P* < 0.01). After treatment with HJSD-H, the expression of cAMP markedly increased (*P* < 0.01). In DG neurons, the expression of cAMP was markedly decreased in the epileptic group compared to that of the control group (*F*_5,444_ = 9.579, *P* < 0.01). After treatment with HJSD-H, the expression of cAMP markedly increased (*P* < 0.01).

**Figure 10:**
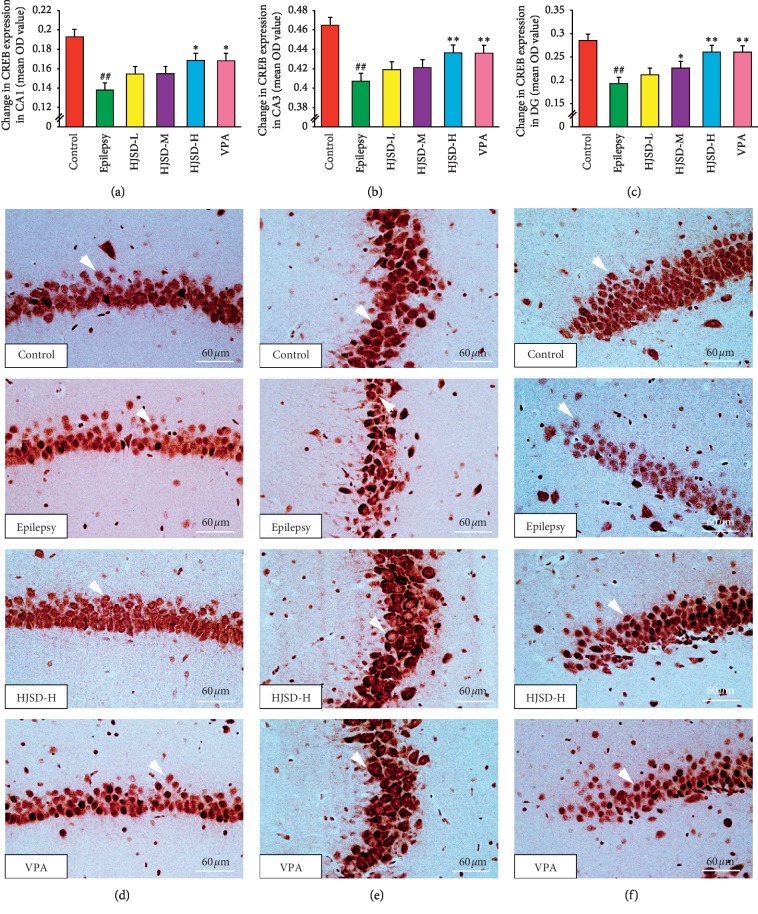
HJSD increases the expression of CREB in rat hippocampal CA1 (a), CA3 (b), and DG (c) regions *in vivo*. Number of CREB-like immunoreactive cells in the hippocampal CA1 (d), CA3 (e), and DG (f) regions. Statistical significance was determined using one-way ANOVA; *n* = 3; ^*∗*^*P* < 0.05, ^*∗∗*^*P* < 0.01, ^##^*P* < 0.01, ^*∗*^Comparison with the epilepsy group; ^#^Comparison with the control group. Positive expression of CREB (arrows) is indicated.

As shown in Figure 10, the expression of CREB in CA1 neurons in the epileptic group was markedly reduced compared to that of the control group (*F*_5,444_ = 3.993, *P* < 0.01). After treatment with HJSD-H, the expression of CREB markedly increased (*P* < 0.05). In CA3 neurons, the expression of CREB was markedly decreased in the epileptic group compared to that of the control group (*F*_5,444_ = 4.855, *P* < 0.01). After treatment with HJSD-H, the expression of CREB markedly increased (*P* < 0.01). In DG neurons, the expression of CREB was markedly decreased in the epileptic group compared to that of the control group (*F*_5,444_ = 9.909, *P* < 0.01). After treatment with HJSD-H, the expression of CREB markedly increased (*P* < 0.01).

**Figure 11:**
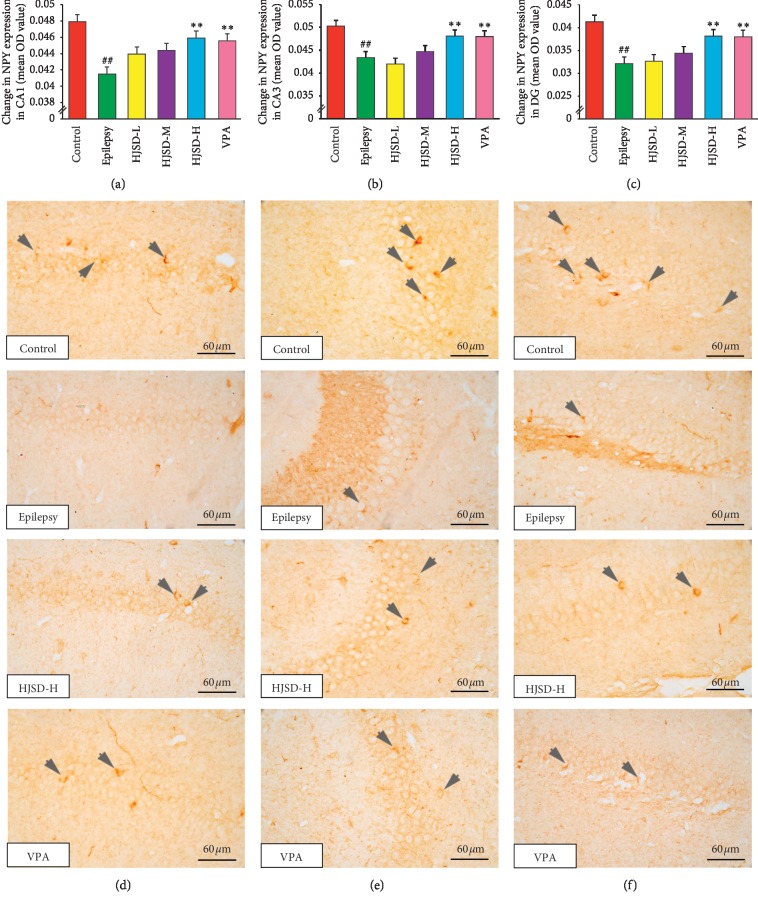
HJSD increases the expression of NPY in rat hippocampal CA1 (a), CA3 (b), and DG (c) regions *in vivo*. Number of NPY-like immunoreactive cells in the hippocampal CA1 (d), CA3 (e), and DG (f) regions. Statistical significance was determined using one-way ANOVA; *n* = 3; ^*∗∗*^*P* < 0.01, ^##^*P* < 0.01, ^*∗*^Comparison with the epilepsy group; ^#^Comparison with the control group. Positive expression of NPY (arrows) is indicated.

As shown in Figure 11, the expression of NPY in CA1 neurons was reduced in the epileptic group compared to that of the controls (*F*_5,444_ = 3.862, *P* < 0.01). After treatment with HJSD-H, the expression of NPY was increased (*P* < 0.01). In CA3 neurons, the expression of NPY in the epileptic group was reduced compared to that of the controls (*F*_5,444_ = 7.252, *P* < 0.01). After treatment with HJSD-H, the expression of NPY was found to be markedly increased (*P* < 0.01). In DG neurons, the expression of NPY in the epileptic group was markedly decreased compared to that of the control group (*F*_5,444_ = 9.189, *P* < 0.01). After treatment with HJSD-H, the expression of NPY markedly increased (*P* < 0.01).

## 4. Discussion

Administration of PTZ resulted in chemical kindling and a chronic and progressive rise in the irritability of the central nervous system, as well as a decrease in the threshold for seizure occurrence [31]. PTZ kindling in rats is a common model for the study of AEDs. Because of the damage it causes to neurons, epilepsy often results in cognitive and emotional behavioral dysfunction [32]. Prolonging the course of epilepsy results in a decline in cognitive abilities and a change in emotional behavior, which becomes more and more obvious.

In this study, a variety of behavioral tests were used to demonstrate the cognitive decline and emotional behavior changes in PTZ-induced epilepsy models. The MWM test is a type of dimensional learning and memorization method directly related to hippocampal function [33, 34], and is a classical cognitive experiment method. The RAM and YM tests reflected the spatial memory and evasive memory of the rats through the analysis of feeding strategies and evasive stimulation [26, 27]. The EPM and OFM tests are commonly used methods to observe the behavioral pharmacological effects of animals, such as fear, anxiety, and alertness [35]. The EPM test stimulates the inherent fear and anxiety of rats followed by their protective emotional response by using the elevated area of the open arm. The OFM test leverages the animal's aversion to exploring an unfamiliar environment, stimulating rats with a new environment to induce anxiety, locomotion, and other behaviors [28]. The OFM test activity index of anticapture reactivity is used to evaluate the effect [36].

Compared with the epilepsy group, the degree of improvement in learning memory and cognitive ability in the HJSD treatment group was significantly improved. In terms of the behavioral performance of rats, the number of the platform crossings increased and the latency of escape shortened in MWM, the residence time of correct feeding arm increased, and the number of reference memory errors decreased in RAM. Also, the number of evasive errors decreased in YM. In addition, the results of this study showed that compared with the epilepsy group, the behavior of the rats in the HJSD treatment group was as follows: in EPM, the time and number of open arms increased; in OFM, the number, time, and distance of entering the center increased and the total distance traveled also increased, which indicates that HJSD may improve the fear and anxiety emotional responses and locomotor abilities of epileptic rats. In these experiments, HJSD played a major role in improving behavioral abnormalities.

HJSD has been used to treat epileptic seizures for many years. Some ingredients in HJSD have been demonstrated to be effective for the treatment of brain damage caused by epilepsy, for example, baicalin (*Scutellaria baicalensis*) and saikosaponin (Radix bupleuri) have significant effects on convulsions, neuroprotective mechanisms, and brain injury caused by epilepsy [14, 15]. According to studies from Liu et al., *α*-Asarone (the Acori graminei rhizoma) has been used to improve various disease conditions including stroke and convulsions [16]. Furthermore, the *Ocimum basilicum* has been shown to be significantly effective in reducing the latency and frequency of seizures [17]. In our preliminary experiments, HJSD was observed to have no toxic effects and no abnormal changes on the nerve cells of normal rats (data not shown). Experimental results showed that HJSD is similar to VPA in preventing convulsions and improving cognitive and affective disorders. Therefore, this study focused on exploring the antiepileptic mechanisms of HJSD and the improvement of cognition and mood deficits that are caused by epilepsy.

Research has shown that the hippocampus is the main area associated with long-term learning and memory, emotion, acousto-optic responses, and taste [37]. It is mainly responsible for the storage, conversion, and orientation of long-term memory, and it has important roles in information processing. Repeated convulsions in rodents have far-reaching and harmful effects on hippocampal long-term memory, which can reduce the plasticity of hippocampal information processing [38]. Hippocampus is the best area to observe the pharmacodynamic changes in cognitive and emotional disorders caused by epileptic injury [39, 40]. The occurrence of epilepsy is closely related to the hippocampus and CA1, CA3, and DG, as important regions of the hippocampus, are important areas of epilepsy research [41–43]. The internal loop of the hippocampus mainly forms three excitatory synaptic connection systems that involve the CA1, CA3, and DG regions, respectively.

cAMP signaling may play a role in epilepsy, this has been known since the 70s; the details, however, remain elusive. The cAMP molecule is generated from ATP by AC when they are stimulated by Gs-linked G protein-coupled receptors (GPCRs). Once formed, cAMP diffuses to act on its effector proteins, of which, cAMP-activated protein kinase (PKA) seems to be the most important (or at least the most studied) in the context of epilepsy, as indicated from the published literature. The role of cAMP signaling can be divided into short-term and long-term effects on epileptogenesis. The short-term effects include the immediate effects, such as channel or receptor phosphorylation, which instantly changes epileptic factors that include neuronal excitability. The long-term effects of cAMP signaling entail protein expression-based changes in epileptogenesis, such as those mediated by CREB. The long-term effects of cAMP signaling also play a major role during epileptogenesis although many of the changes observed are still poorly understood [44]. The CREB is a eukaryotic nuclear protein. It regulates gene transcription. It is one of the key components of cAMP signaling. The brain contains a diverse array of CREB pathways, and Mirza et al. [45] employed a GWAS-based approach to develop the hypothesis that the most influential pathway in focal epilepsy works through AC-cAMP-CREB signaling. Increased CREB phosphorylation has been discovered in both rodent epilepsy models and in humans with treatable and drug-resistant epilepsy [44, 46, 47]. Evidently, targeting the upstream cAMP signals that center on the CREB-mediated transcriptional regulatory machinery may turn out to be a more selective drug target. Considering all of this, it seems peculiar that no drugs targeting the cAMP-generating system have been developed although some might affect the system via an off-target effect. One obvious reason, of course, is the ubiquity and the complexity of cAMP signaling.

Our experimental results have showed that the number of immunoreactive cells of AC, cAMP, and CREB in the epilepsy group is significantly reduced compared with that of the control group. However, conflicting results have also been reported. Some report that there is an increase in the expression of AC or an increase in the level of cAMP in the epilepsy group [48–50]. The difference in these results can perhaps be attributed to the nature of the experimental model used, i.e., acute or chronic [23]. This, however, indicates that the expression of these indicators on immunoreactive cells, and protein tissue expression may have different results in chronic epilepsy. This difference in results may be due to differences in the nature of the model and the degree of response time. Some studies have shown that a loss of AC in mice leads to decreased neuronal activity and depression-like behaviors [51]. In addition, mice lacking the G protein-coupling receptor somatostatin receptor-3 has been reported to show memory deficits in recognition of new objects and reduced levels of cAMP in the brain [52].Our results suggest that HJSD can improve the cognitive function of epileptic rats by increasing the number of immunoreactive cells of AC, cAMP, and CREB, which may be related to the regulation of the AC-cAMP-CREB signaling pathway in hippocampal neurons.

Recently, many researchers believed that NPY has an antiepileptic effect and is important in the physiological regulation of cognitive function in epileptic disease models. NPY plays an important role in depression, anxiety, and other pathological disorders. Its expression is mainly in the limbic system, which is related to emotional responses, especially in the caudal putamen, hippocampus, and other brain tissues of the back [35]. NPY is an endogenous neuropeptide according to Woldbye, and it has a strong antiepileptic function both *in vivo* and *in vitro* [53, 54]. Both in acute and chronic epilepsy models, transgenic NPY results in a remarkable antiepileptic effect when overexpressed in the hippocampus or piriform cortex by rAAV vectors [55, 56].

Our results showed that NPY expression in the epileptic rats was significantly lower than the control group, and the HJSD-H group was significantly higher than that in the epilepsy group. This result showed that HJSD could improve the expression of NPY in the brain of epileptic rats so that it may continue to play an antiepileptic role, and there was no statistical difference in the expression of NPY between the HJSD group and the VPA group. But the results of Vezzani and Sperk [57] showed that NPY expression in epilepsy was higher than in the control group, which was different from our results. We consider that this controversy is related to different methods of epilepsy modeling. However, due to the different research purposes and methods, the results obtained by the researchers are not the same. Seizure suppression by neuropeptide Y in the hippocampus is predominantly mediated by Y2 receptors, which, together with neuropeptide Y, are upregulated after seizures as a compensatory mechanism [58]. Neuropeptide tissue levels often decrease within hours, or 2-3 days after an acute episode of severe seizures [59, 61]. In our study, the epilepsy model was kindled by intraperitoneal injection of PTZ. PTZ is administered chronically at a subconvulsive dose for a number of days, which might be different from the other methods to induce the epileptic model.

This study has provided a mechanism for the antiepileptic effect of HJSD and the improvement of cognitive and emotional disorders by observing hippocampal CA1, CA3, and DG regions, as well as immunohistochemical and behavioral test results. However, further studies are needed to determine the effect of HJSD on the differences (or changes) of the dorsal and ventral hippocampus areas and gene expression can also be used to pinpoint the target of the drug action in detail.

In conclusion, our results demonstrate that HJSD treatment in epileptic rats may significantly inhibit epileptic seizures and improve cognitive and emotional behavior disorders. The antiepileptic effect of HJSD-H was similar to VPA. These experimental results suggest that HJSD might significantly inhibit epileptic seizures by enhancing the AC-cAMP-CREB signaling pathway and NPY expression in the hippocampus. Understanding the regulation of AC-cAMP-CREB signaling and the expression of NPY is expected to elucidate the efficacy of TCM in the treatment of epilepsy and should provide the basis for the development of new Chinese medicines.

## Figures and Tables

**Table 1:** The composition and clinical dosage of HJSD.

English name	Mandarin name	%	g/day
*Scutellaria baicalensis*	Huang qin	20	12
Gynostemma pentaphylla	Jiao gu lan	20	12
Radix bupleuri	Chai hu	20	12
Rhizoma acori graminei	Shi chang pu	15	9
Lotus petiole	He geng	15	9
Basil	Luo le	10	6

## Data Availability

The data used to support the findings of this study are available from the corresponding author upon request.

## Abbreviations

AC: Adenylate cyclase
AEDs: Antiepileptic drugs
ANOVA: One-way analysis of variance
cAMP: Cyclic adenosine monophosphate
CREB: cAMP-response element binding protein
DG: Dentate gyrus
EPM: Elevated plus maze
HJSD: Huazhuo Jiedu Shugan decoction
IP: Intraperitoneal injections
MWM: Morris water maze
NPY: Neuropeptide Y
OFM: Open field maze
PBS: Phosphate-buffered saline
PTZ: Pentylenetetrazol
RAM: Radial arm maze
TCM: Traditional Chinese medicine
VPA: Sodium valproate
YM: Y-maze.

## Supplementary Materials

Supplementary Materials Graphical abstract.
