# Supplementary figures and images for: Effects of Huazhuo Jiedu Shugan Decoction on Cognitive and Emotional Disorders in a Rat Model of Epilepsy: Possible Involvement of AC-cAMP-CREB Signaling and NPY Expression
Source: Evid Based Complement Alternat Med. 2019 Dec 13;2019:4352879. doi: 10.1155/2019/4352879 (PMC6930777; doi:10.1155/2019/4352879)

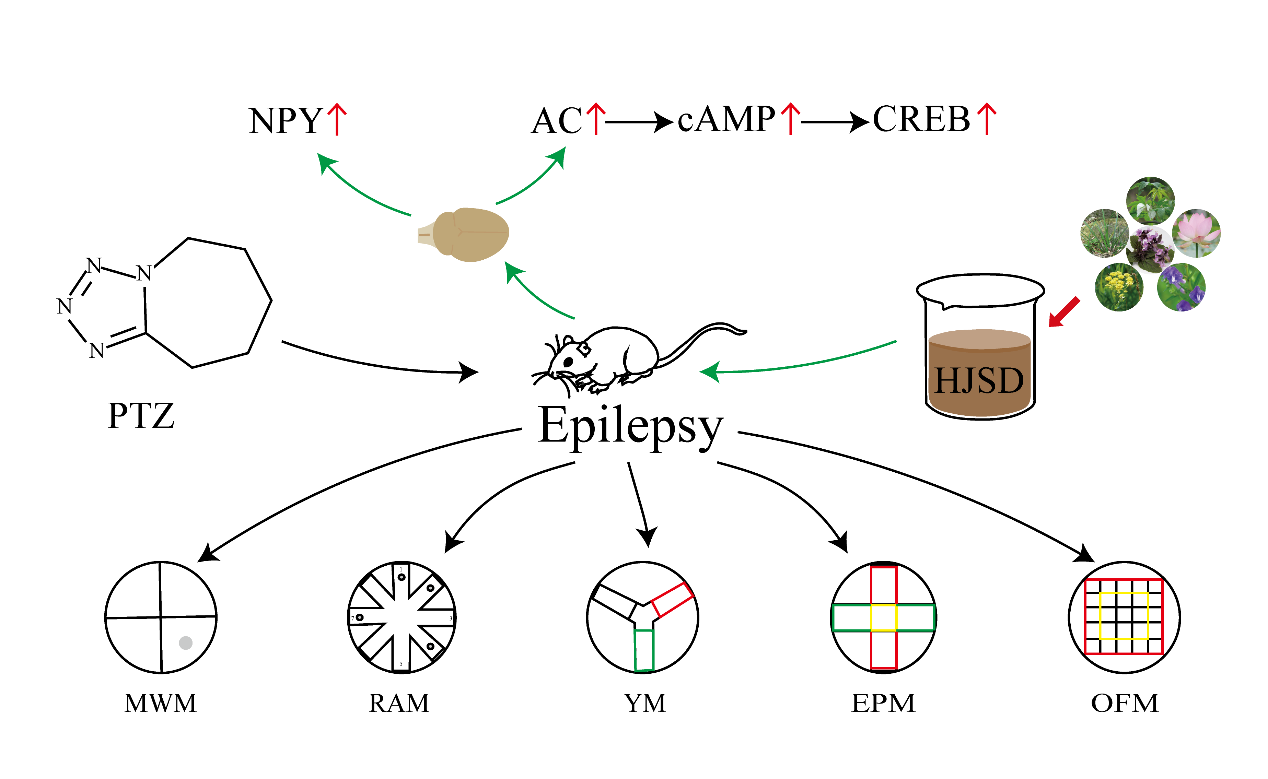

Supplement: Supplementary Materials — Graphical abstract. [file 4352879.f1.docx]
